# Maternal obesity modulates intracellular lipid turnover in the human term placenta

**DOI:** 10.1038/ijo.2016.188

**Published:** 2016-11-22

**Authors:** B Hirschmugl, G Desoye, P Catalano, I Klymiuk, H Scharnagl, S Payr, E Kitzinger, C Schliefsteiner, U Lang, C Wadsack, S Hauguel-de Mouzon

**Affiliations:** 1 Department of Obstetrics and Gynecology, Medical Univeristy of Graz, Auenbruggerplatz 14, Graz, Austria; 2 Department of Reproductive Biology, MetroHealth Medical Center, Case Western Reserve University Cleveland, Cleveland, OH, USA; 3 Core Facility Molecular Biology, Center for Medical Research, Medical University of Graz, Graz, Austria; 4 Clinical Institute of Medical and Chemical Laboratory Diagnostics, Medical University of Graz, Auenbruggerplatz 15, Graz, Austria

## Abstract

**Background:**

Obesity before pregnancy is associated with impaired metabolic status of the mother and the offspring later in life. These adverse effects have been attributed to epigenetic changes *in utero*, but little is known about the role of placental metabolism and its contribution to fetal development.

**Objectives:**

We examined the impact of maternal pre-pregnancy obesity on the expression of genes involved in placental lipid metabolism in lean and obese women.

**Subjects/Methods:**

Seventy-three lean and obese women with healthy pregnancy were recruited at term elective cesarean delivery. Metabolic parameters were measured on maternal venous blood samples. Expression of 88 genes involved in lipid metabolism was measured in whole placenta tissue. Proteins of genes differently expressed in response to maternal obesity were quantified, correlated with maternal parameters and immunolocalized in placenta sections. Isolated primary trophoblasts were used for *in vitro* assays.

**Results:**

Triglyceride (TG) content was increased in placental tissue of obese (1.10, CI 1.04–1.24 mg g^−1^, *P*<0.05) vs lean (0.84, CI 0.72–1.02 mg g^−1^) women. Among target genes examined, six showed positive correlation (*P*<0.05) with maternal pre-pregnancy BMI, namely ATGL (*PNPLA2*), FATP1 (*SLC27A1*), FATP3 (*SLC27A3*), PLIN2, PPARG and CGI-58 (*ABHD5*). CGI-58 protein abundance was twofold higher (*P*<0.001) in placentas of obese vs lean women. CGI-58 protein levels correlated positively with maternal insulin levels and pre-pregnancy body mass index (*R*=0.63, *P*<0.001 and *R*=0.64, *P*<0.001, respectively). CGI-58 and PLIN2 were primarily located in the syncytiotrophoblast and, were upregulated (1.38- and 500-fold, respectively) upon oleic acid and insulin treatment of cultured trophoblast cells.

**Conclusion:**

Pre-gravid obesity significantly modifies the expression of placental genes related to transport and storage of neutral lipids. We propose that the upregulation of CGI-58, a master regulator of TG hydrolysis, contributes to the turnover of intracellular lipids in placenta of obese women, and is tightly regulated by metabolic factors of the mother.

## Introduction

Rates of overweight and obesity have increased dramatically in all regions of the world over the past few decades. One-third of the world's population has a body mass index (BMI) >25 kg m^−^^2^ and the prevalence of obesity (BMI⩾30 kg m^−^^2^) has reached 13% (WHO; 2014). Therefore, it is not surprising that the number of women who are overweight or obese at the time of conception is also increasing. Obese women are predisposed to complications during pregnancy with multiple adverse health outcomes for mothers and their offspring.^1^ Numerous fetal programming and follow-up studies of offspring from obese mothers have documented that these children are at increased risk of diabetes, hypertension and metabolic syndrome later in life.^2, 3, 4^

Fetal growth is strictly dependent on maternal nutrient availability which relies on placental transport of energy substrates from the mother to the fetus. Placenta nutrient transport is dependent on placental size, morphology, transporter capacity/availability and feto-placental blood flow.^5^ Glucose, amino acids, free fatty acids (FFAs) and cholesterol are essential macronutrients for fetal growth, and each nutrient crosses the placenta often through specific transporters. Although glucose is the primary oxidative substrate of fetal tissues, fatty acids (FAs) are critical for fetal brain development and adipose tissue accretion. In maternal circulation, FAs are present in triglycerides (TG), phospholipids (PL) and cholesterol esters (CE). Maternal TGs need to be first broken down into FFAs by placental TG lipases before entering the placenta.^6^ FFAs are then available for uptake into the placenta through FA-transport proteins. Plasma TGs represent the most important source of FA in the human fetus, with about 20–50% of these FAs being derived from maternal circulation.^7^ Several FA-transport proteins, with different affinity for FA chain length and saturation, enable FA uptake and re-esterification in placental cells,^8^ while the mechanisms of FA efflux to the fetal circulation have remained elusive.^7^ Esterified FA can be stored in energy-storage cell organelles, known as lipid droplets (LD).^9^

Evidence from an ovine model suggests that maternal obesity alters placental FA transport through changes in transporter levels rather than TG hydrolysis.^10^ However, in humans there is a paucity of research on the impact of maternal obesity on placental FA uptake with inconsistent information.^11, 12^

Our study aimed to investigate the impact of maternal pre-pregnancy obesity on placental expression of genes involved in FA uptake and metabolism with a special consideration of genes relevant for intracellular lipid storage. We hypothesized that maternal pre-pregnancy obesity impacts lipid storage in the human placenta, which may result in increased availability of FAs for fetal development and growth.

## Materials and Methods

### Study population

Women with a healthy singleton pregnancy (in total *n*=89, placenta biopsies *n*=73) were recruited at term (38–40 weeks of gestation) at the time of elective cesarean delivery. Subjects with multiple gestation, fetal anomalies, intrauterine growth restriction and diabetes (pre-existing and gestational) were excluded. This study was approved by the Institutional Review Board of Metrohealth Medical Center, Case Western Reserve University. Written informed consent was obtained before obtaining blood and placenta tissue. Analysis of placenta tissue biopsies was approved by the ethical review board of the Medical University of Graz (25–401 ex 12/13). Obesity was defined as pre-pregnancy body mass index (BMI)>30 kg m^−^^2^. Maternal pre-pregnancy BMI and metabolic characteristics were obtained from medical records. Maternal blood was collected on admission to labor and delivery, before placement of an intravenous line for hydration. Following double-clamping of the umbilical cord, the placenta was placed in a sterile container. Fragments (~1 cm^3^) of deep placental villous tissue were collected, quickly blotted on gauze and snap-frozen in liquid nitrogen within 10 min of delivery.

### Plasma assays

Plasma glucose was assessed by the glucose oxidase method (Yellow Springs, OH, USA). Plasma insulin concentrations were measured by ELISA (EMD Millipore Corporation, Billerica, MA, USA). Insulin resistance was estimated using homeostasis model assessment homeostatic model assessment insulin resistance (HOMA-IR) as fasting plasma insulin (milliunits per liter) multiplied by fasting plasma glucose ((mg dl^−1^)/405).^13^

Total cholesterol, unesterified cholesterol, TG, PL and FFA were measured using enzymatic reagents (Diasys Diagnostic Systems, Holzheim, Germany) and were calibrated using secondary standards from Roche Diagnostics (Mannheim, Germany). Esterified cholesterol (CE) was calculated as the difference between total and unesterified cholesterol. All measurements were performed on an Olympus AU640 analyzer (Beckman Coulter Inc, Brea, CA, USA). The interassay coefficients of variation were <5%.^14^

### Triglyceride levels in placental tissue

Placental villous tissue (50 mg) was homogenized in 1 ml acetone and total lipids were extracted by overnight incubation on a shaking platform. Samples were centrifuged at 16 000 *g* for 15 min, and 5 μl acetone extract was used to assay the TG concentration by an enzymatic kit (Diasys Diagnostic Systems) following the manufacturer's instructions.

### Quantitative real-time PCR (qRT-PCR) gene expression assays and antibodies for protein analysis

Gene expression assays were purchased from Applied Biosystems (Darmstadt, Germany) and included the following genes: adipose triglyceride lipase ATGL (PNPLA2, gene ID 57104, Taq man assay no. Hs00386101_m1), hormone sensitive lipase, HSL (LIPE, gene ID 3991, Taq man assay no. Hs00193510_m1), α/β hydrolase domain containing 5, CGI-58, (ABHD5, gene ID 51099, Taq man assay no. HS01104373_m1), perilipin 1 (PLIN1, gene ID 5346, Taq man assay no. Hs00160173_m1), perilipin 2, ADRP (PLIN2, gene ID 123, Taq man assay no. HS00605340_m1), perilipin 3, TIP47 (PLIN3, gene ID 10226, Taq man assay no. HS00998416_m1), perilipin 4 (PLIN4, gene ID 729359, Taq man assay no. Hs00287411_m1), perilipin 5, OXPAT (PLIN5, gene ID 440503, Taq man assay no. Hs00965990_m1), TATA box-binding protein (TBP, gene ID 6908, Taq man assay no. Hs00427620_m1).

Western blot membranes were probed with the following antibodies: rabbit anti-adipose triglyceride lipase (ATGL) antibody, 1:1000 (Abcam, Cambridge, UK, cat. no. ab109251), mouse anti-perilipin 2 (ADRP, PLIN2) antibody 1:500 (Progen, Heidelberg, Germany, cat. no. AP 125), mouse anti-perilipin 3 antibody (PLIN3), 1:2000 (R&D Systems, Minneapolis, MN, USA, cat. no. MAB 76641), mouse anti-CGI-58 antibody (ABHD5) 1:250 (Abnova, Taipei, Taiwan, cat. no. H00051099-M01), mouse anti-β-actin antibody 1:20 000 (Abcam, cat. no. ab6276); mouse anti-cyclophilin A (PPIA1) antibody 1:1000 (Abcam, cat. no. ab58144), goat anti-rabbit (horse radish peroxidase conjugated) secondary antibody 1:2000 (Bio-Rad Laboratories, Vienna, Austria, cat. no. 170-6515), goat anti-mouse (horse radish peroxidas conjugated) secondary antibody 1:2000 (Bio-Rad Laboratories, cat. no. 170-6516).

### RNA isolation and quality assessment

Frozen placental tissue (50–100 mg) was homogenized in RLT lysis buffer (Qiagen, Hilden, Germany) by using precellys ceramic kit (Peqlab, Erlangen, Germany) and the MagNA lyser system (Roche). RNA was isolated from placenta tissue homogenates by RNeasy mini kit (Qiagen) following the protocol of the manufacturer. The RNA quality control was performed on a 2100 Bioanalyzer Instrument (Agilent Technologies, Santa Clara, CA, USA) and only samples with a RNA integrity number above 7.0 were considered for analysis.

### mRNA expression analysis by nCounter system (NanoString Technologies)

For gene expression analysis a custom Code Set containing 88 genes was designed at Nanostring Technologies (NanoString Technologies, Seattle, WA, USA). Hybridization of 100 ng total RNA of term placenta was performed in a proof of principle study at Nanostring Headquarters in Seattle according to manufacturer's instructions on a NanoString nCounter Analysis system. Expression data was normalized to 28 validated housekeeping genes (Supplementary Table 1) using the nSolver 2.0 Analysis Software (NanoString Technologies).

### mRNA expression analysis by qRT-PCR

Total RNA was isolated as described above and 2 μg was transcribed into cDNA by using Super Script II reverse transcriptase (ThermoScientific, Waltham, MA, USA) and random hexamer primer (ThermoScientific) following the manufacturer's protocol. qRT-PCR was performed, using Taq man gene expression assays (Applied Biosystem) as indicated above, according to the manufacturer's instructions. A final cDNA concentration of 10 ng per assay was used and measurements were performed in triplicates. The qRT-PCR protocol was: 1 cycle at 95 °C for 10 min; 40 cycles of 95 °C for 15 s; and 60 °C for 1 min and was performed on AB7900 Cycler (Applied Biosystem). mRNA expression of target genes was normalized to TATA box-binding protein (TBP, housekeeping gene), and 2^−ΔCT^ values were used for data representation and statistical analysis if not otherwise stated.

### Cell culture

Primary human trophoblasts were isolated from three different placentas of lean women according to previous published protocol.^15^ Quality control was assessed by immunohistochemistry (cytokeratin 7, vimentin, human HLA -G) and each preparation revealed at least 98% trophoblast cell purity. A total of 3 × 10^6^ cells were seeded in six-well plates and allowed to settle for 24 h. Thereafter, cells were treated for 24 h with 80 μmol l^−1^ oleic acid bound to bovine serum albumin (BSA) (Sigma-Aldrich, Vienna, Austria) alone or in combination with 10 nmol l^−1^ insulin (EMC Milipore Corp., Billerica, MA USA). As control, BSA or BSA in combination with insulin was used in appropriate concentrations. Cells were washed and harvested in laemmli sample buffer (Sigma-Aldrich).

### Immunoblot

Protein extracts from placental tissue were obtained by homogenizing 80–150 mg tissue in RIPA buffer (Sigma-Aldrich) containing protease inhibitor (Roche) on ice. After centrifugation at 13 000 r.p.m., 4 °C for 10 min, the supernatant was withdrawn and protein concentration was determined by BCA protein assay kit (ThermoScientific). For western blot analysis, 10 μg of tissue protein was mixed with laemmli buffer boiled for 5 min at 95 °C and loaded on 10% Tris/Glycine/SDS gels (Bio-Rad Laboratories). After gel electrophoresis, proteins were semi-dry blotted on nitrocellulose membrane (Bio-Rad Laboratories). Membranes were blocked with 5% dry milk (Bio-Rad Laboratories) and incubated with primary antibodies overnight followed by incubation with secondary antibodies and development with chemiluminescence reagent (ThermoScientific). Densidometric analysis was performed using AlphaDigiDoc 1000 (Alpha Innotech Corporation, San Leandro, CA, US). Signals were normalized to β-actin and to the same sample on each blot in order to adjust for inter blot variations.

### Immunohistochemistry

Localization of PLIN2 (1:500) and PLIN3 (1:10 000) was performed on cryostat sections (4 μm), in order to protect the placental lipids from organic solvents. Briefly, cryostat sections were thawed and dried for 30 min at room temperature following fixation with 4% formaldehyde. Between each incubation step slides were washed four times with 88 mM tris-boric acid-EDTA buffer (TBE) containing 0.1% tween (Sigma-Aldrich). For PLIN2 staining, sections were incubated for 10 min with 0.1% Triton X100 in TBE (Fluka, Buchs, Switzerland). Thereafter, primary antibody was added for one hour followed by incubation with primary antibody enhancer (ThermoScientific) for 20 min. Horse radish peroxidas polymer (ThermoScientific) was applied for 30 min in the dark. AEC single solution (ThermoScientific) was used as a substrate (10 min), followed by counter staining with Mayer's haemalaun.

Paraffin-embedded tissue was used for detection of ATLG (1:5000). Paraffin-embedded tissue was cut into 5 μm sections. Sections were treated with Xylol-ethanol for paraffin removal and antigen retrieval in 1 mM EDTA, pH 8.0 and microwaved for 20 min.

### Statistics

The characteristics of the study cohort and the placental data are expressed as mean±s.d. Normal distribution and equal variation of the data was tested. Cohort characteristics were validated by Kruskal–Wallis analysis of variance on ranks followed by Dunn's *post hoc* test. Spearman correlation was performed in order to correlate gene expression data with maternal pre-pregnancy BMI and plasma insulin. Differences between lean and obese group were tested by Mann-Whitney rank sum test. For statistical analyses SPSS statistics, version 22.0 (IBM, Armonk, NY, US) or Sigma Plot, version 12.5 (Systat Software Inc., Erkrath, Germany) were used. The significance level was set at *P*<0.05.

## Results

### Characteristics of the study population

The study population was divided into three groups according to maternal pre-pregnancy BMI. All groups were closely matched for maternal age, gestational age at delivery, parity and blood pressure. As expected, pre-gravid BMI, maternal plasma insulin levels and HOMA-IR index were significantly increased in both obese groups compared with lean group (Table 1). Plasma triglycerides, cholesterol and plasma FFA were similar in the two obese groups and the lean group. Plasma CE and PL were significantly lower in the obese group ⩾35 kg m^−^^2^ in comparison with the lean group. The ratio of birth weight to placental weight was inversely proportional to the pre-pregnancy BMI (Supplementary Table 2).

### Increased triglyceride content in placentas of obese women

TG content was significantly increased in placental tissue of obese in comparison to the lean group (*P*<0.05, Table 1). However, placental TG content was not correlated with maternal pre-gravid BMI (R=0.172, *P*=0.148), total gestational weight gain (R=0.008, *P*=0.948), weight at delivery (R=0.137, *P*=0.25), maternal plasma TG (R=0.110, *P*=0.645) or FFA (R=0.195, *P*=0.410).

### Expression of genes involved in lipid metabolism

Eighty eight genes involved in lipid and FA uptake, storage, metabolism and transfer, were identified through literature search and assessed by using nCounter technology, a target-specific, quantitative gene expression assay (Supplementary Table 1). Out of 34 housekeeping genes validated for their adequacy to the placental study, six genes were regulated by obesity and excluded from further analysis. Target gene expression was then normalized to a panel of 28 not regulated housekeeping genes (Supplementary Table 3). Among the 88 target genes examined, ACAT2 and APOE were negatively correlated with maternal pre-pregnancy BMI (Supplementary Table 1) and six showed significant (*P*<0.05) positive correlation with maternal pre-pregnancy BMI, namely ATGL (*PNPLA2*), FATP1 (*SLC27A1*), FATP3 (*SLC27A3*), PLIN2, PPARG and CGI-58 (*ABHD5*) (Table 2).

### Maternal pre-pregnancy obesity upregulates the ATGL co-activator CGI-58

**Figure 1:**
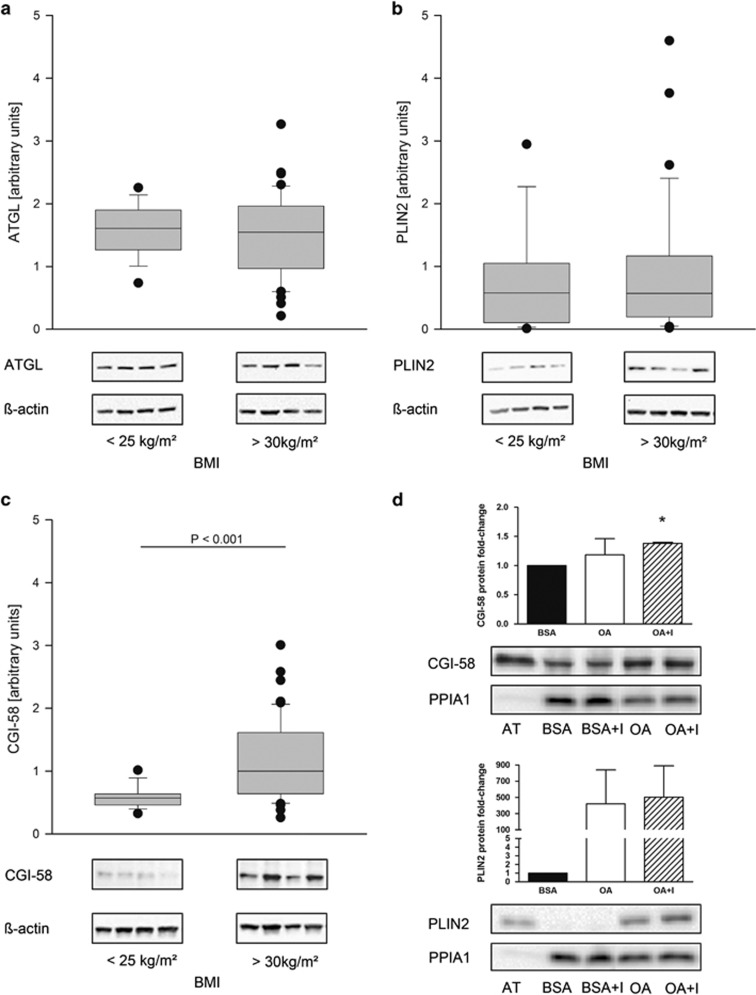
Placental CGI-58 is regulated by maternal pre-pregnancy obesity. Lower panel: representative western blots (*n*=4 per group) of total placental tissue. (**a**) ATGL protein expression. (**b**) PLIN2 protein expression. (**c**) CGI-58 protein expression. All protein signals were quantitated by densitometry, normalized to β-actin as loading control and to one protein sample which was used on each blot to correct for inter blot variations. Sum rank test was performed, differences between the lean (BMI<25 kg m^−2^, *n*=18) and obese (BMI>30 kg m^−2^; *n*=45) group were defined as significant if *P*-values were <0.05. (**d**) Isolated primary trophoblast cells were treated for 24 h with oleic acid (OA, 80 μmol l^−1^), oleic acid and insulin (OA+I, 10nmol l^−1^). Bovine serum albumin (BSA) or BSA and insulin (BSA+I) was used in identical concentrations. Adipose tissue (AT, 2 μg) served as positive control. The upper panel indicates semi-quantitative analysis, signals for BSA were set to 1 and signals for OA and OA+I are presented relative to the BSA control. *P<0.05 indicate statistical significant difference to BSA control. In the lower panel, one representative experiment out of three independent trophoblast isolations is shown.

**Figure 2:**
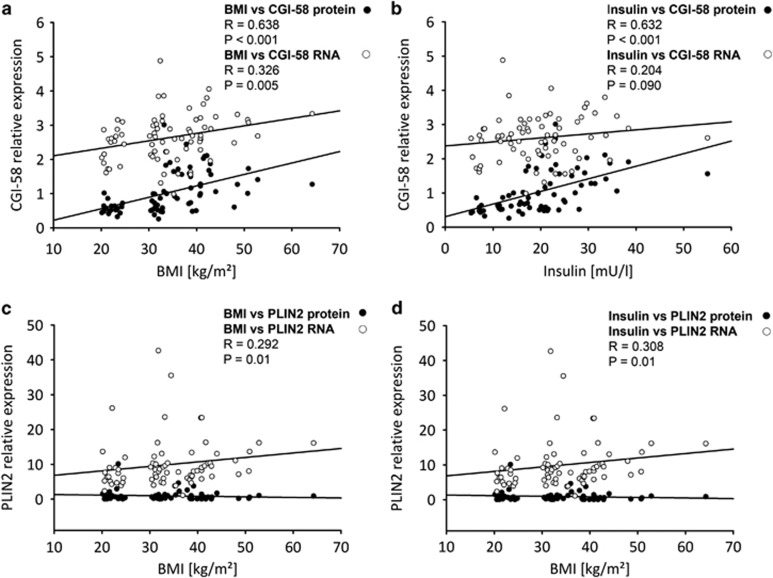
Association of placental CGI-58 with maternal metabolic parameters. Correlation analysis was performed between CGI-58 mRNA or protein expression in placenta tissue and maternal pre-pregnancy BMI (**a**) or maternal plasma insulin (**b**). PLIN2 mRNA and protein levels were correlated with (**c**) maternal pre-pregnancy BMI and maternal plasma insulin levels (**d**). Black circles (•) protein expression on thr open circles (○) mRNA expression. Spearman correlation was defined as significant if *P*-values were <0.05.

ATGL and PLIN2 protein expression was similar in placentas of lean and obese groups. (Figures 1a and b) CGI-58 protein abundance was significantly higher (*P*<0.001) in placentas of obese vs lean women (Figure 1c). Furthermore, CGI-58 mRNA and protein expression correlated positively with maternal BMI (Figure 2a) and maternal insulin levels (Figure 2b). PLIN2 correlated with maternal BMI (Figure 2c) and maternal insulin (Figure 2d) but only at the mRNA level. In isolated primary trophoblast cells CGI-58 and PLIN2 protein levels were upregulated (by 1.38- and 500-fold, respectively) in the presence of oleic acid and insulin in the culture medium (Figure 1d).

### ATGL and PLIN2 are localized in the syncytiotrophoblast

**Figure 3:**
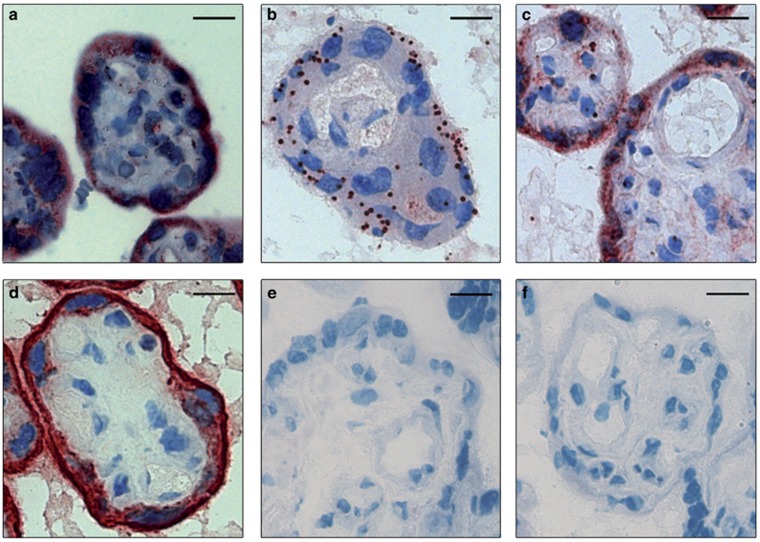
Localization of ATGL, PLIN2 and PLIN3 in term placenta tissue. (**a**) ATGL was localized exclusively in the syncytiotrophoblast layer. (**b**) PLIN2 was detectable on the syncytiotrophoblast by a clear punctate staining. (**c**) PLIN3 was mainly localized in the syncytiotrophoblast. (**d**) Cytokeratin 7 was used as positive control for the syncytiotrophoblast layer. Negative control staining for rabbit IgG (**e**) and mouse IgG (**f**) was performed with equal IgG concentrations. Scale bar, 50 μm.

ATGL was primarily localized in the syncytiotrophoblast and punctate staining was observed in the fetal endothelium (Figure 3a). PLIN2 showed a dot-like staining pattern in the syncytiotrophoblast layer and some cells of the villous stroma (Figure 3b). PLIN3, the other PLIN isoform expressed in the human placenta, was exclusively localized as continuous staining in the syncytiotrophoblast (Figure 3c; Supplementary Figure 1 and 2). Cytokeratin 7 was used as positive control for the syncytiotrophoblast layer (Figure 3d). Rabbit IgG (Figure 3e) and mouse IgG (Figure 3f) were used as negative controls. Despite repeated efforts with the antibody used in western blots, CGI-58 was not detectable by IHC.

## Discussion

Gestational adjustments in lipid homeostasis, coupled to anatomical and physiological changes, support placental and fetal growth while maintaining maternal energy balance. We report for the first time that maternal obesity is associated with increased content in placental TGs despite similar circulating maternal TG in lean and obese groups. Whereas there are discrepancies regarding whether plasma TG levels are modified in obese pregnant women,^11, 16^ our data are consistent with elevated total lipid content reported in the placenta of obese women.^17^ These results suggest that obesity in general, but not maternal TG levels may account for the lipid accumulation in the placenta by either an increased FA hydrolysis,^18^ or by changes of the intracellular lipid compartmentalization.

We identified six genes, strongly involved in TG metabolism, (FATP1, FATP3, PPARG, PLIN2, ATGL, CGI-58) showing a positive correlation with maternal pre-gravid BMI. FA transport protein FATP1 and FATP3, are members of the solute carrier family 27 (SLC27A), which are important membrane proteins for cellular FA uptake.^19^ The function and specificity of FATP1-6 is currently under debate primarily due to the lack of specific antibodies for this group of membrane proteins.^11, 20, 21^ The location of FATP1 on the apical microvillous membrane of the placental syncytium suggests that this isoform facilitates FA uptake from the maternal circulation.^11, 22^

To further characterize placental pathways for lipid storage and mobilization we focused on PLIN2, ATGL and CGI-58, which showed a strong positive correlation with maternal pre-gravid obesity. Perilipin 2 (PLIN2 or adipophilin, ABHD5) is a member of the perilipin protein family, which coat intracellular LDs.^23^ The punctate immunostaining in the syncytiotrophoblast layer was similar to the pattern previously reported in placental fetal membranes^24^ and the amnion epithelium.^25^ Incubation of primary human trophoblast cells with insulin and oleic acid enhanced the expression of PLIN2 suggesting that the formation of placental LDs is regulated by insulin and long chain FAs.^23^ Furthermore, the similar PLIN2 content in lean and obese placentas suggests that there is an active turnover of neutral lipids in the syncytium and/or a fast proteasomal degradation of unbound perilipin 2.^26^

Adipose triglyceride lipase (ATGL, former desnutrin) is a LD-associated enzyme which hydrolyzes FA from TG stores.^27, 28, 29^ In the adipose tissue, ATGL is responsible for maintaining basal lipolytic activity and can be activated by its master regulator CGI-58.^30, 31^ A role of CGI-58 in obesity has been suggested by the observation that CGI-58 knockdown mouse model does not develop high-fat diet induced obesity.^32^ Beside its role as co-activator of TG hydrolysis, the proposed lysophosphatidic acid acyltransferase activity of CGI-58 may generate lipid mediators involved in insulin signaling and thereby obesity, a role which is currently under debate.^33, 34, 35, 36, 37, 38^ The concomitant increase in placental TG content and CGI-58 expression in placentas of obese women suggests a regulatory role for CGI-58 in placental lipid turnover, which might be under influence of elevated insulin levels in the obese mother. This concept is further supported by the positive correlation between ATGL, CGI-58 and maternal pre-gravid BMI. To exclude the contribution of other potential causes of placental changes throughout pregnancy we further tested possible relation of maternal body weight at term, total gestational weight gain and placental TG levels. The lack of any correlations of these latte parameters, further support the association between maternal pre-pregnancy metabolism and placental TG composition.

Although the results are novel and a human model was implemented which is definitely a major strength of this study, there are some limitations worth mentioning. First, pregnancy is characterized by profound temporal modifications in the women's metabolism and the placenta is also exposed to spatio-temporal changes from both, the maternal and fetal circulation during pregnancy. Although the results of our study only compare one single time point before pregnancy maternal confounders such as weight at delivery and total weight gain were excluded. Second, the study describes associations of maternal pre-pregnancy BMI with placental changes, but does not provide evidence that these changes are directly physiologically relevant. However, growing evidence in the literature suggests that maternal pre-pregnancy obesity appeared to be at a greater risk of developing adverse pregnancy outcomes.^39^ A potential direct effect by pre-pregnancy BMI of women should be addressed in future by prospectively designed follow-up studies.

In conclusion, our data demonstrate that maternal obesity modulates the expression of lipid-related genes in the human placenta. The coordinated regulation of LD-associated proteins by the metabolic state of the mother highlights for the first time the importance of the placental lipolytic machinery in the regulation of excess lipid stores. Specifically, the tight control of placental CGI-58 by the metabolic state of the mother may contribute to the regulation of feto-placental lipid pathways. The strong associations between maternal obesity and placental lipid homeostasis call for future studies to directly establish its causal relationship.

## Figures and Tables

**Table 1:** Characteristics of the study subjects values are expressed as mean (±s.d.)

	*BMI⩽25 kg m^−^*^*2*^	*BMI 30–34.9 kg m^−^*^*2*^	*BMI*⩾*35 kg m^−^*^*2*^
	*(*n=*34)*	*(*n=*23)*	*(*n=*32)*
Pre-pregnancy BMI (kg m^−^^2^)	22.2 (±1.5)	32 (±1.2)***	42 (±6.1)***
Weight gain (kg)	16.4 (±6.0)	12.2 (±6.3)	12.2 (±9.2)**
Age (years)	29.4 (±6.6)	29.6 (±7.2)	26.2 (±5.4)
GA at delivery (weeks)	39.2 (±0.4)	39.2 (±0.5)	39.3 (±0.5)
Smoker (%)	17	43	24
Insulin (mU l^−1^)	9.6 (±3.2)	19.1 (±7.1)***	25 (±8.2)***
Insulin resistance (HOMA-IR)	1.8 (±0.7)	3.6 (±1.3)***	5.2 (±2.5)***
Parity	1.4 (±0.9)	1.6 (±0.9)	1.8 (±1.0)
Ethnicity (aa/cauc/hisp) (%)	(13/82/5)	(26/70/4)	(50/44/6)
Blood pressure systolic	119 (±13)	118 (±12)	117 (±11)
Placenta tissue TG (mg g^−1^)	0.87 (±0.3)	1.16 (±0.32)*	1.12 (±0.40)*
	*BMI⩽25 kg m^−^^2^*	*BMI 30–34.9 kg m^−^^2^*	*BMI*⩾*35 kg m^−^^2^*
*Maternal plasma lipids*	*(*n=*17)*	*(*n=*9)*	*(*n=*9)*
TG (mg dl^−1^)	167 (±79)	168 (±71)	130 (±53)
Cholesterol (mg dl^−1^)	214 (±50)	197 (±63)	160 (±51)
CE (mg dl^−1^)	143 (±35)	129 (±41)	104 (±33)*
PL (mg dl^−1^)	247 (±50)	232 (±59)	185 (±41)*
FFA (mmol l^−1^)	0.86 (±0.24)	0.98 (±0.22)	0.88 (±0.18)

Abbreviations: aa, African American; ANOVA, analysis of variance; BMI, body mass index; cauc, Caucasian; hisp, Hispanic; HOMA-IR, homeostatic model assessment-insulin resistance; TG, triglycerides; CE, cholesterol ester; PL, phospholipids; FFA, free fatty acids.

Non-parametric ANOVA on Ranks (Kruskal–Wallis) was performed followed by Dunn's *post hoc* test. Differences between the lean and distinct obese groups were indicated as *P<0.05, **P<0.01 or ***P<0.001, respectively.

**Table 2:** Effect of maternal obesity on genes implicated in placental lipid storage

*Gene*	*Gene expression by nCounter*	
	*Spearman correlation*	P*-value*
ATGL	0.248	0.034
CGI-58	0.326	0.005#
FATP1	0.238	0.042
FATP3	0.245	0.037
PLIN2	0.258	0.028
PPARG	0.278	0.017

Abbreviations: BMI, body mass index; qRT-PCR, quantitative real-time PCR.

Target-specific gene expression analysis in placental tissue biopsies (n=73) was performed by nCounter technology or qRT-PCR (#). Maternal pre-pregnancy BMI ranged between 20–64 kg m^−^^2^. Spearman correlation was performed between target genes and maternal pre-pregnancy BMI. *P*-values<0.05 were defined as statistical significant.
