# Supplementary material for: Maternal obesity modulates intracellular lipid turnover in the human term placenta
Source: Int J Obes (Lond). 2016 Nov 22;41(2):317–23. doi: 10.1038/ijo.2016.188 (PMC5309341; doi:10.1038/ijo.2016.188)
Supplement: Supplementary Material [file ijo2016188x1.docx]

**Supplementary Table 1: Genes involved in placental lipid uptake, storage and metabolism.**

|  |  |  |  | **Nanostring** | | **Validation by qRT-PCR** | |
| --- | --- | --- | --- | --- | --- | --- | --- |
|  | **Lipid, FA binding/uptake; lipoproteins** | **Gene** | **Gene ID** | **Spearman** | **P-value** | **Spearman** | **P-value** |
| 1 | LDL receptor | LDLR | 3949 | -0.025 | 0.837 |  |  |
| 2 | VLVL receptor | VLDLR | 7436 | -0.049 | 0.680 |  |  |
| 3 | Scavenger receptor class B, member 1 (SRB1) | SCARB1 | 949 | 0.198 | 0.094 |  |  |
| 4 | Fatty acid transport protein 1, FATP1 | SLC27A1 | 376497 | 0.238 | 0.042 |  |  |
| 5 | Fatty acid transport protein 2, FATP2 | SLC27A2 | 11001 | 0.005 | 0.965 |  |  |
| 6 | Fatty acid transport protein 3, FATP3 | SLC27A3 | 11000 | 0.245 | 0.037 |  |  |
| 7 | Fatty acid transport protein 4, FATP4 | SLC27A4 | 10999 | 0.144 | 0.225 |  |  |
| 8 | Fatty acid transport protein 5, FATP5 | SLC27A5 | 10998 | n.d. | n.d. |  |  |
| 9 | Fatty acid transport protein 6, FATP6 | SLC27A6 | 28965 | -0.001 | 0.996 |  |  |
| 10 | CD36 molecule, fatty acid translocase, FAT | CD36 | 948 | -0.115 | 0.333 |  |  |
| 11 | Glutamic-oxaloacetic transaminase 2, mitochondrial | GOT2 | 2806 | 0.202 | 0.087 |  |  |
| 12 | Fatty acid binding protein 1, L-FABP | FABP1 | 2168 | n.d. | n.d. |  |  |
| 13 | Fatty acid binding protein 3, H-FABP | FABP3 | 2170 | n.d. | n.d. |  |  |
| 14 | Fatty acid binding protein 4, A-FABP | FABP4 | 2167 | n.d. | n.d. |  |  |
| 15 | Fatty acid binding protein 7, B-FABP | FABP7 | 2173 | n.d. | n.d. |  |  |
| 16 | Glycosylphosphatidylinositol anchored high density lipoprotein binding protein 1 | GPIHBP1 | 338328 | n.d. | n.d. |  |  |
| 17 | Apolipoprotein A-1 | APOA1 | 335 | n.d. | n.d. |  |  |
| 18 | Apolipoprotein C2 | APOC2 | 344 | n.d. | n.d. |  |  |
| 19 | Apolipoprotein C3 | APOC3 | 345 | n.d. | n.d. |  |  |
| 20 | Apolipoprotein A4 | APOA4 | 337 | n.d. | n.d. |  |  |
| 21 | Apolipoprotein A5 | APOA5 | 116519 | n.d. | n.d. |  |  |
| 22 | Apolipoprotein E | APOE | 348 | -0.243 | 0.038 |  |  |
| 23 | Apolipoprotein M | APOM | 55937 | n.d. | n.d. |  |  |
| 24 | Sphingosine-1-phosphate receptor 1 | S1PR1 | 1901 | 0.240 | 0.041 |  |  |
| 25 | Sphingosine-1-phosphate receptor 3 | S1PR3 | 1903 | -0.039 | 0.741 |  |  |
| 26 | ATP-binding cassette subfamily A member 1 | ABCA1 | 19 | 0.067 | 0.572 |  |  |
| 27 | ATP-binding cassette subfamily G member 1 | ABCG1 | 9619 | -0.072 | 0.545 |  |  |
|  | **Lipid storage/esterification** | **Gene** | **Gene ID** | **Spearman** | **P-value** | **Spearman** | **P-value** |
| 28 | Perilipin 1 | PLIN1 | 5346 | n.d. | n.d. |  |  |
| 29 | Perilipin 2, adipophilin, ADRP | PLIN2 | 123 | 0.258 | 0.028 | 0.292 | 0.013 |
| 30 | Perilipin 3, TIP47 | PLIN3 | 10226 | n.e. | n.e. | 0.098 | 0.407 |
| 31 | Perlilipin 5, OXPAT | PLIN5 | 440503 | n.d. | n.d. |  |  |
| 32 | α/β hydrolase domain containing 5, CGI-58 | ABHD5 | 51099 | -0.167 | 0.158 | 0.326 | 0.005 |
| 33 | Fat mass and obesity associated | FTO | 79068 | -0.014 | 0.906 |  |  |
| 34 | Lipoprotein lipase | LPL | 4023 | -0.088 | 0.458 | -0.032 | 0.786 |
| 35 | Endothelial lipase, EL | LIPG | 9388 | 0.074 | 0.531 | 0.020 | 0.867 |
| 36 | Hepatic lipase, HL | LIPC | 3990 | n.d. | n.d. | -0.004 | 0.970 |
| 37 | Adipose triglyceride lipase, ATGL | PNPLA2 | 57104 | 0.248 | 0.034 | -0.158 | 0.184 |
| 38 | Monoacylglycerol lipase | MGLL | 11343 | 0.065 | 0.583 |  |  |
| 39 | Diacylglycerol lipase alpha | DAGLA | 747 | n.d. | n.d. |  |  |
| 40 | Diacylglycerol lipase beta | DAGLB | 221955 | 0.218 | 0.064 |  |  |
| 41 | Hormone sensitive lipase, HSL | LIPE | 3991 | n.d. | n.d. | -0.019 | 0.873 |
| 42 | Phospholipase A2 group 7 | PLA2G7 | 7941 | n.d. | n.d. |  |  |
| 43 | Phospholipase A2 group 2A | PLA2G2A | 5320 | -0.161 | 0.175 |  |  |
| 44 | Platelet activating factor receptor | PAFR | 5724 | -0.161 | 0.174 |  |  |
| 45 | Cholesterol ester hydrolase | CES1 | 1066 | -0.085 | 0.475 |  |  |
|  | **FA elongation/desaturation/oxidation** | **Gene** | **Gene ID** | **Spearman** | **P-value** | **Spearman** | **P-value** |
| 46 | ELOVL fatty acid elongase 1 | ELOVL1 | 64834 | -0.159 | 0.178 |  |  |
| 47 | ELOVL fatty acid elongase 2 | ELOVL2 | 54898 | -0.041 | 0.729 |  |  |
| 48 | ELOVL fatty acid elongase 3 | ELOVL3 | 83401 | n.d. | n.d. |  |  |
| 49 | ELOVL fatty acid elongase 4 | ELOVL4 | 6785 | n.d. | n.d. |  |  |
| 50 | ELOVL fatty acid elongase 5 | ELOVL5 | 60481 | n.d. | n.d. |  |  |
| 51 | ELOVL fatty acid elongase 6 | ELOVL6 | 79071 | n.d. | n.d. |  |  |
| 52 | ELOVL fatty acid elongase 7 | ELOVL7 | 79993 | 0.031 | 0.795 |  |  |
| 53 | Stearoyl-CoA desaturase, Delta-9-desaturase | SCD | 6319 | -0.070 | 0.559 |  |  |
| 54 | Fatty acid desaturase 1 | FADS1 | 3992 | -0.048 | 0.685 |  |  |
| 55 | Carnitine palmitoyltransferase 1A (liver) | CPT1A | 1374 | -0.119 | 0.316 |  |  |
| 56 | Carnitine palmitoyltransferase 1B (muscle) | CTP1B | 1375 | n.d. | n.d. |  |  |
| 57 | Carnitine palmitoyltransferase 1C (brain) | CTP1C | 126129 | n.d. | n.d. |  |  |
| 58 | Carnitine palmitoyltransferase 2 | CPT II | 1376 | 0.085 | 0.476 |  |  |
| 59 | Acyl-CoA-Synthase long-chain family member 3 | ACSL3 | 2181 | -0.008 | 0.949 |  |  |
| 60 | Acyl-CoA-Synthase long-chain family member 5 | ACSL5 | 51703 | 0.035 | 0.766 |  |  |
| 61 | Acetyl-CoA acyltransferase 2 | ACAA2 | 10449 | 0.068 | 0.568 |  |  |
|  | **Cholesterol and Steroid Hormone Biosynthesis** | **Gene** | **Gene ID** | **Spearman** | **P-value** | **Spearman** | **P-value** |
| 62 | Acetyl-CoA acetyltransferase 1 | ACAT1 | 38 | -0.150 | 0.204 |  |  |
| 63 | Acetyl-CoA acetyltransferase 2 | ACAT2 | 39 | -0.234 | 0.046 |  |  |
| 64 | Sterol O-acyltransferase 1 | SOAT1 | 6646 | n.e. | n.e. | 0.076 | 0.521 |
| 65 | Sterol O-acyltransferase 2 | SOAT2 | 8435 | n.e. | n.e. | n. d. | n. d. |
| 66 | 3-hydroxy-3-methylglutaryl-CoA synthase 1 | HMGCS1 | 3157 | 0.033 | 0.783 |  |  |
| 67 | 3-hydroxy-3-methylglutaryl-CoA reductase | HMGCR | 3156 | 0.025 | 0.832 |  |  |
| 68 | Lanosterol synthase | LSS | 4047 | -0.043 | 0.717 |  |  |
| 69 | Squalene epoxidase | SQLE | 6713 | -0.102 | 0.390 |  |  |
| 70 | Methylsterol monooxygenase 1, SC4MOL | MSMO1 | 6307 | -0.126 | 0.288 |  |  |
| 71 | NAD(P) dependent steroid dehydrogenase-like | NSDHL | 50814 | -0.059 | 0.618 |  |  |
| 72 | Emopamil binding protein | EBP | 10682 | 0.042 | 0.726 |  |  |
| 73 | Cytochrome P450, family 51, subfamily A, polypeptide 1 | CYP51A1 | 1595 | 0.085 | 0.472 |  |  |
| 74 | Cytochrome P450, family 1, subfamily A, polypeptide 1 | CYP1A1 | 1543 | n.d. | n.d. |  |  |
| 75 | Hydroxysteroid-17β-dehydrogenase 1 | HSD17B1 | 3292 | -0.220 | 0.062 |  |  |
|  | **Hormons; Transcription factors** | **Gene** | **Gene ID** | **Spearman** | **P-value** | **Spearman** | **P-value** |
| 76 | Leptin | LEP | 3952 | n.d. | n.d. |  |  |
| 77 | Leptin receptor | LEPR | 3953 | -0.007 | 0.950 |  |  |
| 78 | Adiponectin | ADIPOQ | 9370 | n.d. | n.d. |  |  |
| 79 | Adoponectin receptor1 | ADIPOR1 | 51094 | 0.033 | 0.783 |  |  |
| 80 | Adoponectin receptor2 | ADIPOR2 | 79602 | -0.112 | 0.347 |  |  |
| 81 | Peroxisome proliferator-activated receptor alpha | PPARA | 5465 | -0.061 | 0.609 |  |  |
| 82 | Peroxisome proliferator-activated receptor gama | PPARG | 5468 | 0.277 | 0.018 |  |  |
| 83 | Peroxisome proliferator-activated receptor delta (beta) | PPARD | 5467 | -0.025 | 0.833 |  |  |
| 84 | sterol regulatory element binding transcription factor 1, SREBP1 | SREBF1 | 6720 | 0.204 | 0.084 |  |  |
| 85 | Sterol regulatory element binding transcription factor 2, SREBP2 | SREBF2 | 6721 | 0.188 | 0.112 |  |  |
| 86 | cAMP responsive element binding protein 1 | CREB1 | 1385 | -0.047 | 0.692 |  |  |
| 87 | cAMP responsive element binding protein 3 | CREB3 | 10488 | -0.066 | 0.577 |  |  |
| 88 | cAMP responsive element binding protein 5 | CREB5 | 9586 | 0.014 | 0.905 |  |  |

In total expression of 88 genes was examined in placental tissue specimens from women with pre-pregnancy BMI between 20 – 64 kg/m². Validation of selected genes was performed by qRT-PCR. Spearman correlation between gene expression and maternal BMI was performed and p-values < 0.05 were defined as statistical significant. n.d.: not detected, n.e.: not examined.

**Supplementary Table 2: Characteristics of the neonates.**

|  | BMI ≤ 25 kg/m^2^ | | BMI 30 – 34.9 kg/m^2^ | | BMI 35 – 39.9 kg/m^2^ | | BMI ≥ 40 kg/m^2^ | | Statistics |
| --- | --- | --- | --- | --- | --- | --- | --- | --- | --- |
| Birth weight (kg) | 3.32 | (± 0.49) | 3.24 | (± 0.44) | 3.33 | (± 0.44) | 3.28 | (± 0.50) | n.s. |
| Birth length (cm) | 49.8 | (± 2.0) | 48.6 | (± 1.4) | 49.2 | (± 1.4) | 49.0 | (± 2.0) | n.s. |
| Body fat mass (%) | 11.7 | (± 3.5) | 11.9 | (± 3.3) | 13.0 | (± 3.0) | 13.2 | (± 4.0) | n.s. |
| Placental weight (g) | 580 | (± 166) | 684 | (± 233) | 601 | (± 139) | 701 | (± 144) | n.s. (P=0.055) |
| Infant/placental weight ratio | 5.8 | (± 1.1) | 5.0 | (± 1.1)* | 5.7 | (± 0.9) | 4.8 | (± 0.8)* | P<0.01 |
| Number of cases | 36 | | 23 | | 14 | | 18 | |  |

Pregnant women (n=89), with singleton pregnancies and gestational age (GA) ≥ 38 weeks were included. Neonatal characteristics are expressed as mean (± SD) P-values < 0.05 were defined as significantly different between groups. *significantly different to control group (BMI ≤25kg/m²); n.s.: not significant

**Supplementary Table 3: Housekeeping genes suitable for placental gene expression analysis in maternal obesity.**

|  | **Housekeeping gene (HKG)** | **Gene ID** | **Suitable HKG** | **ANOVA P-value** |
| --- | --- | --- | --- | --- |
| 1 | ANGEL1 | 23357 | yes | 0.447 |
| 2 | ATG4B | 23192 | yes | 0.093 |
| 3 | C7orf26 | 79034 | yes | 0.844 |
| 4 | CLTC | NM_004859.2 | yes | 0.212 |
| 5 | CYC1 | 1537 | yes | 0.143 |
| 6 | GAPDH | NM_002046.3 | yes | 0.559 |
| 7 | GUSB | NM_000181.1 | no | 0.007 |
| 8 | HAUS2 | 55142 | yes | 0.694 |
| 9 | HPRT1 | 3251 | no | 0.048 |
| 10 | KCTD2 | 23510 | yes | 0.137 |
| 11 | KIAA2013 | 90231 | yes | 0.782 |
| 12 | NEK9 | 91754 | yes | 0.379 |
| 13 | OAZ1 | 4946 | yes | 0.385 |
| 14 | PAIP1 | 10605 | yes | 0.872 |
| 15 | PGK1 | NM_000291.2 | yes | 0.796 |
| 16 | POLDIP3 | 84271 | no | 0.055 |
| 17 | PPIA | 5478 | yes | 0.875 |
| 18 | PPP1R10 | 5514 | yes | 0.906 |
| 19 | RING1 | 6015 | yes | 0.100 |
| 20 | RNF220 | 55182 | yes | 0.626 |
| 21 | RPL30 | 6156 | yes | 0.205 |
| 22 | SDHA | 6389 | no | 0.000 |
| 23 | SMARCD1 | 6602 | yes | 0.274 |
| 24 | TBP | 6908 | yes | 0.667 |
| 25 | TNIP2 | 79155 | no | 0.081 |
| 26 | TOP1 | 7150 | yes | 0.559 |
| 27 | TRADD | 8717 | yes | 0.389 |
| 28 | TUBB | NM_178014.2 | yes | 0.446 |
| 29 | UNC45A | 55898 | yes | 0.334 |
| 30 | VPS18 | 57617 | yes | 0.318 |
| 31 | WDR45L | 56270 | yes | 0.621 |
| 32 | YWHAZ | 7534 | yes | 0.557 |
| 33 | ZC3H10 | 84872 | no | 0.082 |
| 34 | ZNF101 | 94039 | yes | 0.615 |

34 housekeeping genes were examined in placental tissue of women with pre-pregnancy BMI between 20 – 64 kg/m². HKGs with significantly different or close significance p-values (0.05 – 0.09) were excluded from the normalization process. P-values < 0.05 were defined as statistical significant.

P = 0.042

PLIN1

PLIN2

PLIN3

PLIN4

PLIN5

**Supplementary Figure 1: PLIN2 and PLIN3 mRNA is expressed in the placenta.** mRNA expression was normalized to TBP (housekeeping gene) and displayed as non-logarithmic values, differences between the lean (BMI < 25 kg/m², n=18) and obese group (BMI > 30 kg/m², n=55) were tested by Sum rank test and significance set at p- < 0.05.


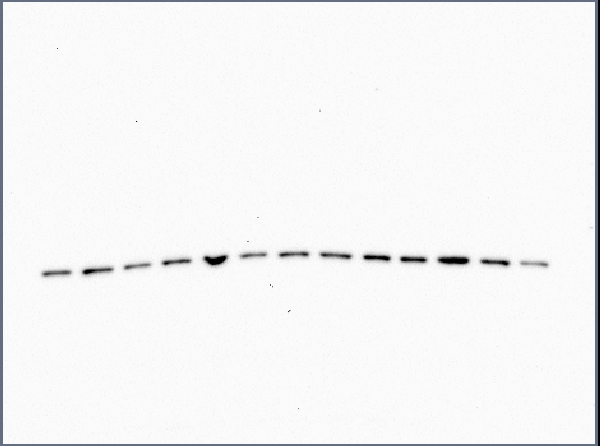


PLIN3

GAPDH

< 25 kg/m²


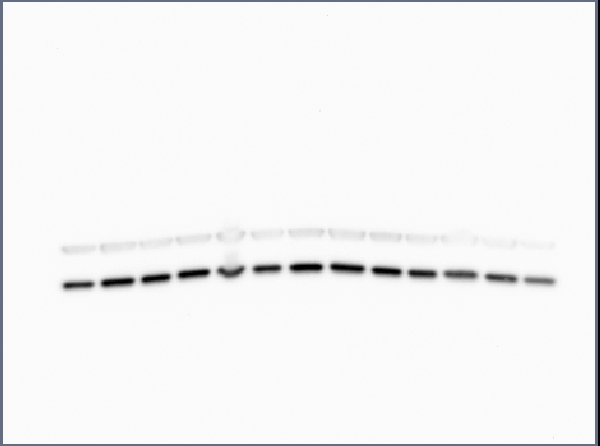

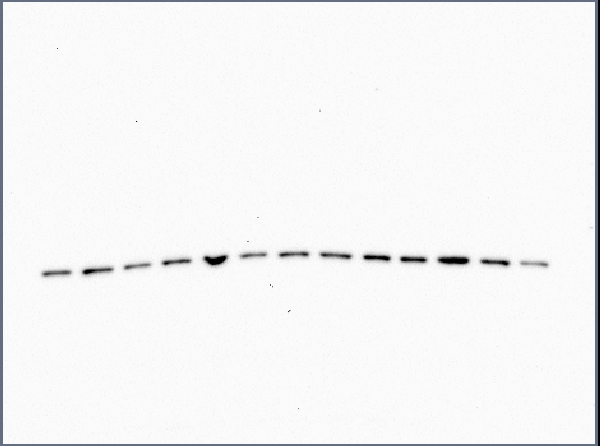

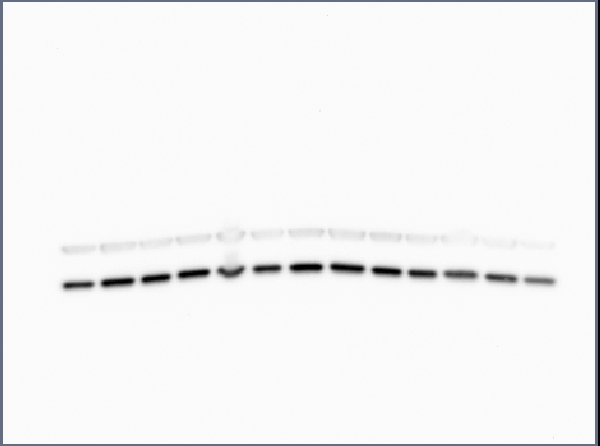


> 30 kg/m²

BMI

Supplementary Figure 2: PLIN3 protein expression in placenta. Representative western blot (n=4 per group) are demonstrated in the lower panel. All protein signals were determined by densitometry, normalized to GAPDH as loading control and one sample as a control for inter blot variation. Sum rank test was performed, differences between the lean (BMI < 25 kg/m², n=18) and obese (BMI > 30 kg/m²; n=45) group were defined as significant if p-values were < 0.05.
